# Improving the yield of circulating tumour cells facilitates molecular characterisation and recognition of discordant HER2 amplification in breast cancer

**DOI:** 10.1038/sj.bjc.6605676

**Published:** 2010-05-11

**Authors:** L M Flores, D W Kindelberger, A H Ligon, M Capelletti, M Fiorentino, M Loda, E S Cibas, P A Jänne, I E Krop

**Affiliations:** 1 Department of Medical Oncology, Dana-Farber Cancer Institute, Boston, MA, USA; 2 Department of Biology, University of Massachusetts, Boston, MA, USA; 3 Harvard Medical School, Boston, MA, USA; 4 Center for Molecular Oncologic Pathology, Dana-Farber Cancer Institute, Boston, MA, USA; 5 Department of Pathology, Brigham and Women's Hospital, Boston, MA, USA

**Keywords:** circulating tumour cells, breast cancer, lung cancer, HER2, FISH

## Abstract

**Background:**

Circulating tumour cells (CTCs) offer a non-invasive approach to obtain and characterise metastatic tumour cells, but their usefulness has been limited by low CTC yields from conventional isolation methods.

**Methods:**

To improve CTC yields and facilitate their molecular characterisation we compared the Food and Drug Administration-approved CellSearch Epithelial Kit (CEK) to a simplified CTC capture method, CellSearch Profile Kit (CPK), on paired blood samples from patients with metastatic breast (*n*=75) and lung (*n*=71) cancer. Molecular markers including Human Epidermal growth factor Receptor 2 (HER2) were evaluated on CTCs by fluorescence *in situ* hybridisation (FISH) and compared to patients’ primary and metastatic cancer.

**Results:**

The median cell count from patients with breast cancer using the CPK was 117 *vs* 4 for CEK (*P*<0.0001). Lung cancer samples were similar; CPK: 145 cells *vs* CEK:4 cells (*P*<0.0001). Recovered CTCs were relatively pure (60–70%) and were evaluable by FISH and immunofluorescence. A total of 10 of 30 (33%) breast cancer patients with HER2-negative primary and metastatic tissue had HER2-amplified CTCs.

**Conclusion:**

The CPK method provides a high yield of relatively pure CTCs, facilitating their molecular characterisation. Circulating tumour cells obtained using CPK technology demonstrate that significant discordance exists between HER2 amplification of a patient's CTCs and that of the primary and metastatic tumour.

The need for access to samples of metastatic cancer and the inherent difficulty in obtaining these using conventional biopsies, has led to interest in alternative sources of metastatic cells. One such alternative is to analyse circulating tumour cells (CTCs). Circulating tumour cells are thought to be quite rare in the blood of most patients with metastatic disease – it has been estimated that they are present at a frequency of 1 in 10^9^ blood cells (Mostert *et al*, 2009). Circulating tumour cells provide the potential ability to monitor metastatic disease, prognostic information and a means to perform non-invasive molecular interrogation of cancers, as these cells can provide a molecular snapshot, or real-time biopsy, of tumour cells. Circulating tumour cells can also potentially be used to determine the mechanisms of acquired resistance to targeted therapies, which only occur or evolve during the course of drug treatment. The ability to isolate a relatively large number of pure CTCs is necessary for these downstream applications.

Circulating tumour cells have become increasingly accepted as both an independent prognostic factor and as a marker of therapeutic response and attempts have been made to standardise their isolation and characterisation (Hayes and Smerage, 2008). The automated CellSearch System (Veridex; Warren, NJ, USA) is the first method to be clinically approved by the United States Food and Drug Administration to capture and detect CTCs. This system relies on immunomagnetic isolation followed by the use of fluorescence microscopy for analysis. Two different but related methods of sample preparation, the CellSearch Epithelial Cell Kit (CEK) and the CellSearch Profile Kit (CPK) are currently available. Both methods use a positive immunomagnetic selection with anti-epithelial cell adhesion molecule antibodies linked to iron particles to enrich for CTCs. In the CEK system, captured cells are permeabilised and labelled with cytokeratin and CD45-specific antibodies, and the nuclear stain 4′-6-diamidino-2-phenylindole (DAPI), and are analysed by semi-automated counting of appropriately labelled CTCs (Riethdorf *et al*, 2007). In contrast, the CPK procedure does not involve labelling or enumerating the cells, but rather only uses the anti-epithelial cell adhesion molecule antibody coupled to iron particles to yield an enriched population of CTCs, which can be used for molecular analyses. However, in contrast to the CEK method, the CPK procedure is not Food and Drug Administration approved for clinical management.

The primary limitation of the CEK system is the low yield of CTCs. In several large clinical trials of patients with metastatic breast cancer, the median number of isolated CTCs was five per 7.5 ml of blood (Budd *et al*, 2006; Hayes *et al*, 2006; Hayes and Smerage, 2008). Although the low numbers of CTCs are sufficient to provide clinical prognostic information, in which a cutoff of ⩾5 CTCs per 7.5 ml of blood is used to delineate a poor prognosis population, the ability to perform molecular analyses of the CTCs is limited. In addition, it has been reported that the degree of leukocyte contamination using the CEK system is quite high, which further complicates molecular analyses of CTCs. These limitations have led to the development of alternative CTC isolation methods, including technologies based on size filtration, density gradients, and microfluidic techniques, which have demonstrated increased yields of CTCs (Vona *et al*, 2000; Nagrath *et al*, 2007; Adams *et al*, 2008; Williams *et al*, 2009). For example, a microfluidic chip-based CTC technology has been developed, which isolates relatively high numbers of CTCs and can be used to non-invasively detect drug-sensitive and -resistant epidermal growth factor receptor (EGFR) mutations from non-small cell lung cancer (NSCLC) patients (Nagrath *et al*, 2007; Maheswaran *et al*, 2008). This method uses epithelial cell adhesion molecule-specific antibodies that are conjugated to posts within the CTC chip. Currently this system is limited by the lack of widespread availability of the system, the inability to release the CTCs from the microfluidic posts, prolonged processing time (samples are run at 1–2 ml h^–1^), and the requirement that samples be processed within a few hours of collection (Nagrath *et al*, 2007).

To address some of the limitations of current CTC systems, we evaluated the CPK system as a method to isolate and study CTCs. Unexpectedly, this method resulted in isolation of >20-fold more CTCs than the CEK method. We further characterised the purity of the isolated CTCs and evaluated them using immunofluorescence (IF) and fluorescence *in situ* hybridisation (FISH). Our findings suggest that a modification in the use of the current CellSearch system can result in a significant increase in the ability to isolate and characterise CTCs from patients with breast cancer and NSCLC.

## Materials and methods

### Patients

Between December 2007 and March 2009 patients with metastatic NSCLC (*n*=71) or metastatic breast cancer (*n*=75) were identified from the Thoracic Oncology and Women's Cancer clinics at the Dana Farber Cancer Institute. Characteristics of the patients are described in the Supplementary Table S1. The blood was collected from each donor into CellSave blood collection tubes (Veridex) or ethylenediaminetetraacetic acid (EDTA) tubes, where specified. The blood samples were maintained at room temperature and processed within a maximum of 72 h after collection. All samples from patients with breast cancer were obtained at the time they were initiating a new therapy for the treatment of their disease. All of the samples from patients with NSCLC were obtained while the patient was currently under therapy. Control blood samples were drawn from healthy volunteers with no history of malignant disease. All patients provided written informed consent, and studies were approved by the Dana Farber Cancer Institute Institutional Review Board (IRB).

### Sample processing and counting

Samples were processed using the CEK method, including semi-automated counting, according to the manufacture's instructions (Johnson & Johnson, 2008). Samples processed using the CPK method (per manufacture's instructions, (Johnson & Johnson, 2008)) were collected in a dilution buffer (phosphate buffered saline, 0.5% bovine serum albumin and 0.1% sodium azide) and immediately transferred to glass slides as cytospin preparations using a ThermoFisher Cytospin 3. The centrifugation was performed at 500 **g** for 5 min with a cytology funnel, thin filter (Thermo Fisher Scientific, Pittsburgh, PA, USA), and a SuperFrost Plus slide (Thermo Fischer Scientific). The cells on the resulting cytospin slide were fixed with methanol for IF or immunohistochemistry or fixed with methanol:acetic acid (3 : 1) for FISH. Cells were stained with anti-cytokeratin phycoerythrin/DAPI/CD45-allophycocyanin (Dako, Carpinteria, CA, USA) and then manually counted using a standard fluorescence microscope (Olympus, Center Valley, PA, USA). Cytokeratin staining was considered positive when cells displayed unequivocal cytoplasmic cytokeratin staining in a ring-like pattern (confirming an intact cytoplasm) surrounding an intact nucleus.

### Cell lines and reagents

The breast cancer cell line SK-BR-3 was obtained from American Type Culture Collection. The EGFR-mutant (del E746_A750) NSCLC cell line, HCC827, has been described previously (Mukohara *et al*, 2005). The two cell lines were grown in Dulbecco's Modified Eagle Medium or Roswell Park Memorial Institute 1640 (CellGrowth, Invitrogen, Life Technologies, Carlsbad, CA, USA) respectively, supplemented with 10% foetal bovine serum. Gefitinib and lapatinib were obtained from commercial sources. Stock solutions of both drugs were prepared in dimethyl sulphoxide and stored at −20°C. For *in vitro* recovery experiments SKBR3 cells were grown in DMEM with 10% foetal bovine serum, 10 *μ*g ml^–1^ EGF, and 1.5 mM L-glutamine. The HCC287 cells were grown in ACL4 (Invitrogen, Life Technologies) with 10% foetal bovine serum. For detailed methods see Supplementary methods.

### Fluorescence *in situ* hybridisation (FISH)

The EGFR/CEP 7 and Human Epidermal growth factor Receptor 2 (HER2)/Centromere Probe 17 (CEP17) bacterial artificial chromosome (BAC) probes were obtained from Vysis Molecular (Abbot Park, IL, USA). BAC mesenchymal-epithelial transition factor (MET) (RP11-95I20) was obtained from CHORI (Children Oakland Research Hospital, Oakland, CA, USA). Labelling was done with the Nick Translation Kit (Vysis, Abbott Molecular, Des Plaines, IL, USA). See Supplementary data for detailed methods of FISH analysis of CTC, and for IF and immunohistochemistry staining methods.

## Results

### The CPK method isolates a greater number of cells than the CEK method

**Figure 1:**
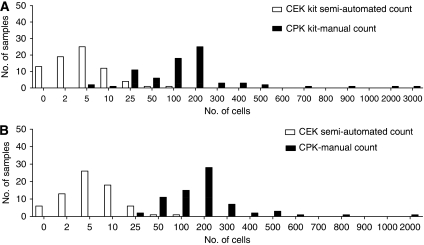
CPK method improves cell yields over CEK method. The blood samples from patients with (**A**) breast cancer (*n*=75) or (**B**) NSCLC (*n*=71) processed in parallel by the CEK method with semi-automated quantification (open columns) or the CPK method with manual quantification (closed columns).

We hypothesised that CTCs may be lost in the additional permeabilisation, labelling, and wash steps specific to the CEK method, particularly if CTCs are more ‘fragile’ than other cell types. To directly compare the yield of cells isolated using CEK and CPK procedures, we collected two peripheral blood samples from patients with metastatic breast cancer (*n*=75) and NSCLC (*n*=71), and processed them in parallel. Cells obtained using the CPK method were counted manually and were compared with the standard CEK counts obtained using the CellSearch system. The median cell count from patients with breast cancer using the CPK method was 116.5 per 7.5 ml of blood (range 4–2432) compared with 4 (range 0–57) using the CEK method (*P*<0.0001, Figure 1A). The median cell count from patients with NSCLC processed using the CPK method was 145 per 7.5 ml of blood (range 5–1801) compared with 4 (range 0–53) using the CEK procedure (*P*<0.0001; Figure 1B). We also compared these findings on a per patient basis (Supplementary Figures S1A and B) and demonstrated that for all patients, the cell count by the CPK method exceeds the cell count by the CEK method (breast cancer: *R*^2^=0.004919; lung cancer: *R*^2^=0.006952). To determine whether the observed yield differences between the CEK and CPK methods are due to differences in the method of cell counting (manual *vs* semi-automatic), we compared CTC counts obtained using the automated CEK method with that of a manual cell count of the same sample. The manual CEK count was performed by removing the enumerated samples from the ‘MagNest’ cartridges, processing them as cytospin preparations and counting them manually. In patients (*n*=75 patients; 100 samples) with breast cancer (Supplementary Figure S1E), the median cell count using the semi-automated CEK method was 4 per 7.5 ml of blood (range 0–57) compared with 5 per 7.5 ml of blood (range 0–61) when the CEK processed specimens were counted manually (*P*=NS). Similarly, in NSCLC patients (*n*=71 patients; 100 samples), the median cell count using the semi-automated CEK method was four per 7.5 ml of blood (range 0–53) compared with five per 7.5 ml of blood (range 5–67) when the CEK processed specimens were counted manually (*P*=not significant; Supplementary Figure S1F). We further compared these findings on a per patient basis (Supplementary Figures S1C and S1D) and observed a strong correlation between the cell counts obtained using the semi-automated CEK method with the manual CEK method (breast cancer specimens: *R*^2^=0.9930; lung cancer specimens: *R*^2^=0.7200). Together, our findings suggest that the CPK method isolates a greater number of cells than the CEK method and that these findings are not due to differences in the method of cell counting.

### The CPK method isolates a highly enriched population of CTCs

**Figure 2:**
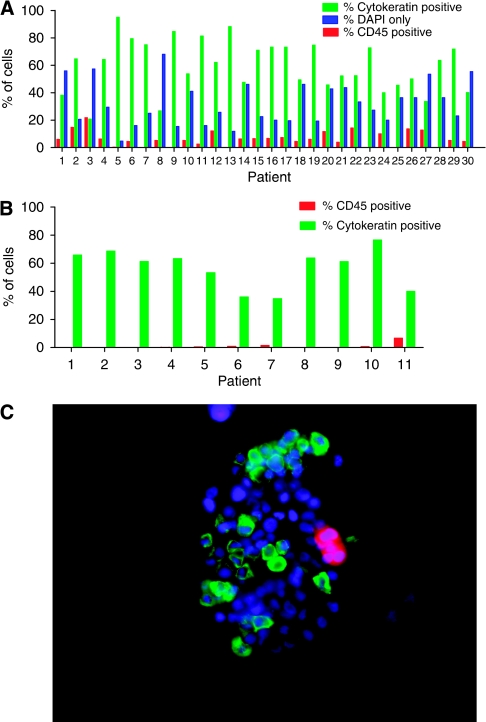
The CPK method isolates a highly enriched population of CTCs. Percentage of total cells captured by the CPK method from patients with NSCLC, staining for cytokeratin (AE1/AE3), CD45, or DAPI nuclear stain alone by (**A**) immunofluorescence or (**B**) FACS. Similar results were seen with samples from patients with breast cancer. (**C**) Representative immunofluorescence image of CPK-captured cells from patient with NSCLC, labelled with cytokeratin (green), DAPI (blue), and CD45 (red).

One explanation for the improved yields observed with the CPK method is that the isolated cells are not all CTCs but instead a mixture of CTCs and contaminating leukocytes. This determination is critical if the isolated cells are to be used for any subsequent analyses. To determine the composition of the cells isolated using the CPK method, we performed IF using anti-CD45 and anti-cytokeratin (AE1/AE3) antibodies in 30 NSCLC and 30 breast cancer patients. Three distinct cell populations were observed: (1) CD45 positive, cytokeratin negative, (2) CD45 negative, cytokeratin positive, and (3) cells that were negative for both CD45 and cytokeratin but stained with the nuclear stain DAPI (Figure 2C). Approximately 60% of cells isolated by CPK (range: 21–95%) were identified as tumour cells by cytokeratin staining, whereas 6% (range: 1–22%) were identified as white blood cells (WBCs) by CD45 staining (Figure 2A and data not shown). These percentages correspond to a median absolute number of cytokeratin positive cells of 280 per sample (range 20–20 000) and a median of 34 (range 20–476) CD45-positive cells per sample. The remaining 32% (range: 5–68%) of cells stained with DAPI only and could not be classified as either tumour cells or leukocytes. As a complementary approach, we used fluorescence-activated cell sorting on 11 patients (eight NSCLC and three breast cancer) to compare the populations of CTCs with WBCs (Figure 2B). The results were similar for cytokeratin (mean positive: 61% range 35–77%), except the frequency of WBCs was estimated to be even lower (mean positive: 1% range 0–7%) than by IF (compare Figure 2A and B).

**Figure 3:**
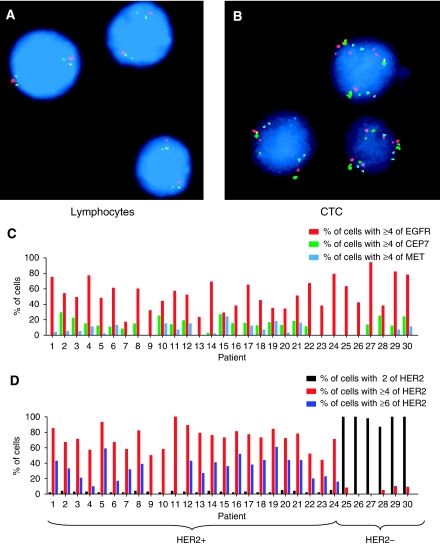
FISH analysis confirms that samples processed by the CPK method have a low percentage of contaminating normal cells. Representative FISH images of cells processed by CPK method, (**A**) lymphocyte with two copies of CEP7 (green), EGFR (red), and MET (blue), (**B**) CTC with amplified EGFR and MET. (**C**) Percentage of total cells captured by the CPK method from patients with NSCLC with ⩾4 copies of EGFR, CEP7, and MET per nucleus. (**D**) Percentage of total cells captured by the CPK method from patients with clinically defined HER2-positive (patient number 1–24) or HER2-negative (patient number 25–30) breast cancer, with the indicated copies of HER2 per nucleus.

As an additional method to evaluate the nature of the cells isolated using the CPK method, we performed FISH using probes for *HER2*, *EGFR*, and *MET*. Given that a mean of 32% of cells had denuded membranes (DAPI-only cells; Figure 2A) and thus could not be definitely stained for cytokeratin, the identification of aneuploidy and/or gene amplification in a high fraction of the cells would strongly suggest that some or all of these cells were CTCs and not WBCs (Figures 3A and B). Among the CTCs from patients with breast cancers clinically defined as HER2 positive from analyses of their primary tumours (FISH+ and/or IHC 3+ by local testing, *n*=24), 72% of all captured cells had a *HER2* gene copy number ⩾4. This threshold has been used as an indicator of HER2 amplification in previous studies (Meng *et al*, 2004). Only 3% of the cells had two copies of *HER2*. In an additional six patients with clinically defined HER2-negative breast cancers, only 5% of captured cells had ⩾4 copies of HER2 (Figure 3D). In contrast, the majority (95%) of cells in those patients had a *HER2* gene copy number of 2 (Figure 3D). Similarly, among samples from NSCLC patients (*n*=30), 53% had ⩾4 copies of EGFR and 13% had ⩾4 copies CEP 7 (Figure 3C). Only a minority of cells (5%) had two copies of EGFR and CEP 7.

As an additional control, we analysed 40 samples from healthy volunteers with no history of malignant disease. No tumour cells were found with either the CEK semi-automated enumeration or with the CPK manual count (Supplementary Figure S2A). In samples processed using the CPK method, the median total number of cells was five per 7.5 ml of blood (range: 0–50). These cells were positive for CD45 and negative for cytokeratin using IF, with only two gene copies of *CEP7*/*EGFR* or *HER2* as detected by FISH analysis, consistent with normal WBC. Collectively, these data indicate that that majority of cells captured by the CPK system are in fact CTCs with only a minority of cells being WBCs.

All of the samples described were prepared using the low-speed cytospin method using a single cytology funnel with thin filter (see Materials and Methods section). We also evaluated other techniques for preparing slides, including high-speed cytospin, different funnel types, a thin prep or cell block, and applying the samples to slides directly as a smear. In a comparative analysis, the low-speed cytospin method produced the highest yield, with the least contamination, least cell damage, and the least slide-to-slide variation in comparison to other various methods (data not shown).

### The CPK method has low intrapatient variability and samples are stable for at least 72 h

We next examined whether the number of cells isolated from patients with cancer using the CPK method was reproducible and stable over time. We first obtained three samples from each of seven patients with NSCLC at the same time point and processed them in parallel. The number of cells isolated was similar (Supplementary Figure S3C) with a low coefficient of variability (CV: 9.7%). We also evaluated the impact of time on the ability to isolate CTCs using the CPK method. As many clinical trials involve multiple centres, it is important to determine the stability of unprocessed samples to determine whether the yield of CTCs declines over time. For these studies, we collected three CellSave (LLC a Johnson&Johnson company, Raritan, NJ, USA) or EDTA tubes from seven NSCLC patients and incubated the samples at room temperature for 24–72 h before processing by the CPK method. We specifically evaluated the collection of blood into both CellSave and EDTA tubes to determine the impact of the fixative (present in the CellSave tubes; absent in the EDTA tubes) on the impact of cell recovery over time. We isolated similar numbers of cells using both the CellSave (Supplementary Figure S3A) and EDTA tubes (Supplementary Figure S3B). There was no significant decline (coefficient of variation (CV) 9.1% and CV 6.5%) in the number of cells isolated with either method over the 72-h period and no decline in the number of cells isolated from CellSave tubes incubated at room temperature for up to 144 h (Supplementary Figure S3A and data not shown). However, there was significant decline in the number of cells isolated from EDTA tubes incubated at room temperature for ⩾96 h before processing by the CPK method.

### Treatment affects yield of cell recovery

**Figure 4:**
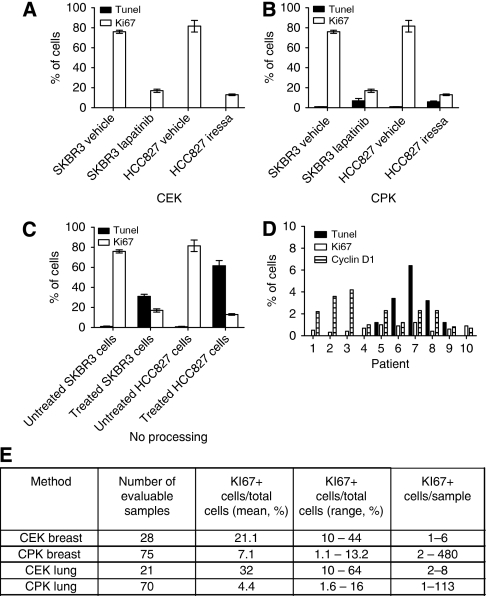
Apoptotic CTCs are less effectively captured by CEK or CPK methods. SKBR3 (HER2^+^ breast cancer) or HCC827 (EGFR mutant NSCLC) cells treated with vehicle or tyrosine kinase inhibitor (SKBR3: 1 *μ*M lapatinib and HCC827; 1 *μ*M gefitinib) for 24 h and processed with (**A**) CEK method. (**B**) CPK method or (**C**) smeared directly on slide without processing. Plots depict percentage of cells (±1 s.d.) staining for Ki67 (open bars, proliferation marker) or TUNEL (closed bars, apoptosis marker) by immunohistochemistry. (**D**) Percentage of CPK-processed CTCs from patients with NSCLC staining positive for KI67 and TUNEL. (**E**) Comparison of KI67 expression in CTCs recovered by CEK or CPK methods.

Previous studies have found that when tumour cell lines are spiked into blood and isolated using the CEK method, the rate of cell recovery is high (85–95% Riethdorf *et al*, 2007). However, the actual CTC yields from cancer patients using the CEK method are limited (Sleijfer *et al*, 2007). We hypothesised that one explanation for the low yields seen in clinical practice is that CTCs are more ‘fragile’ than cancer cell lines either due to apoptosis or damage acquired during transit through the bloodstream and, therefore, are not captured as efficiently with the anti-epithelial cell adhesion molecule antibodies and/or are lost in the post-capture washing and labelling steps. To investigate this possibility, we examined breast (SKBR3; *HER2* amplified) and lung cancer (HCC827; *EGFR* del E746_750) cell lines either unprocessed or spiked into normal blood, then processed with the CPK or CEK methods. Cells were pretreated with the EGFR/HER2 inhibitor lapatinib (SKBR3) or the EGFR inhibitor gefitinib (HCC827) to induce apoptosis, or were untreated. The cells were then stained with Ki67 or Apoptosis Detection Using Terminal Transferase and Biotin-16-dUTP (TUNEL) to evaluate proliferating and apoptotic cells, respectively. As expected, unprocessed cells demonstrated increased rates of apoptosis and decreased proliferation when treated with inhibitor (Figure 4C). Interestingly, no apoptotic cells were noted in the samples processed with the CEK method in either untreated or treated samples, although the treated cells that were captured by that method did have decreased levels of Ki67 staining, consistent with the effect of the inhibitor (Figure 4A). In samples processed with the CPK method, a small percentage of TUNEL-positive cells were recovered in the untreated samples, which increased with inhibitor treatment, although not to the level of unprocessed samples (Figure 4B).

We also evaluated the frequency of Ki67- and TUNEL-staining cells isolated from seven NSCLC patients using the CPK method. The frequency of TUNEL-staining cells was low (mean 1.9% range 0–6.4%) similar to what was observed in the SKBR3 and NSCLC cell lines (Figure 4D). The frequency of Ki67-staining cells was also lower (mean 0.7% range 0.3–1.2%) in the CTCs from cancer patients compared with the levels seen with cell lines (Figure 4D). When parallel samples were processed using the CEK method, no TUNEL-positive cells were detected, but the fraction of Ki67-positive cells was numerically higher than in the CPK processed samples (Figure 4E). This observation is consistent with the hypothesis that potentially more ‘fragile’ apoptotic and non-proliferating cells are disproportionably lost by the more processing intensive CEK method. However, because of the small number of cells recovered, it was not possible to make a definitive comparison. Together with the data obtained from the cell lines, these findings suggest that CTCs undergoing apoptosis in the blood stream, either spontaneously or as result of treatment effect, may be too fragile to survive the capture step and/or are being eliminated during subsequent specimen processing. This effect seems to be more pronounced with the CEK than CPK method and may help explain the increased CTC yields seen with the latter technique. These observations may also help to explain why alternative CTC-capture techniques, such as the microfluidic chip or microfiltration systems that minimise the number of labelling and wash steps are associated with an increase in CTC yield over the CEK method.

### Characterisation of HER2 amplification of CTCs by FISH analysis

Several small studies have reported varying degrees of discordance between the HER2 status of a patient's breast cancer CTCs and that of their primary tumour (Meng *et al*, 2004; Wulfing *et al*, 2006; Pestrin *et al*, 2009). No data exists examining the relationship between the CTCs’ HER2 status and that of metastatic tissue. We hypothesised that the ability of the CPK technology to provide relatively high numbers of CTCs and allow robust FISH analysis would facilitate the determination of the relationship between the HER2 status of a patients primary breast cancer, metastatic lesions, and CTCs. To test this hypothesis, we assessed the presence of HER2 amplification by FISH, normalised for CEP 17, on CTCs from 75 women with breast cancer for whom the HER2 status of the primary cancer and a metastatic biopsy sample was available (patient characteristics available in Supplementary Table S1).

For patients with *HER2* gene amplification of their primary breast cancer, the degree of concordance with the HER2 status of their CTCs was high, with only 1 of 45 (2%) patients demonstrating loss of *HER2* amplification in the corresponding CTCs (Table 1). In that one patient with discordance, the metastatic biopsy was HER2 amplified, reflecting the primary cancer. In contrast, for those patients in whom the primary breast cancer was HER2 negative, significant discordance between the primary, metastatic biopsy, and CTC was observed. A total of 10 (33%) of these 30 patients with HER2-negative primary cancers had HER2-positive CTCs by FISH analysis. The median HER2/CEP17 ratio of the CTCs in these patients was 7.1. Interestingly, in 9 of the 10 patients with discordance, the metastatic biopsy specimen was HER2 negative.

### Evaluation of biologic properties of CTCs isolated using the CPK method

#### Expression of receptor tyrosine kinases

The CPK method is able to isolate a highly enriched population of CTCs and greater numbers of CTCs than the CEK method. These findings provide the opportunity to initiate the molecular characterisation of CTCs. In addition to FISH (Figure 3), we evaluated the membrane expression of total and phosphorylated HER2 and EGFR using IF on the breast and lung cancer cell lines spiked into blood and recovered with the CPK (Supplementary Figure S4A). The IF analysis was superior to chromogenic immunohistochemical staining, as the immunohistochemical substrate tended to bind non-specifically to ferroparticles, and resulted in high background staining (data not shown). We evaluated the expression of EGFR and pEGFR from CTCs in seven NSCLC patients (Supplementary Figure S4C). We were able to detect staining in all samples although the percent of cells staining for either EGFR or pEGFR was lower than in the HCC827 cells that had been spiked into blood and processed using the CPK method. We also used IF to evaluate membrane staining of EGFR, pEGFR, HER2 and pHER in CTCs from 20 patients with clinically HER2-positive breast cancer (Supplementary Figure S5B). Similar to the CTCs isolated from NSCLC patients, we were able to detect EGFR, pEGFR, HER2, and pHER using IF in the CTCs and the frequency of staining was less than in the SKBR3 tumour cell line (Supplementary Figure S5B).

#### *In vitro* propagation

The standard CellSave tubes contain a fixative that prevents the isolation of viable tumour cells. Having observed that CTCs can be efficiently isolated from the blood drawn in EDTA tubes that do not contain a fixative (Supplementary Figure S3B), we explored whether we could isolate SKBR3 and HCC827 cells using EDTA tubes and grow them *in vitro*. We spiked a range (5–1000) of SKBR3 or HCC827 cells into the normal blood drawn into EDTA tubes and processed them using the CPK method. We were able to isolate viable SKBR3 and HCC827 cells (Supplementary Figure S5) and propagate them *in vitro*. However, this required a minimum input of 50 cells to be processed (Supplementary Figure S5). Optimal growth occurred in the presence of enriched medium and cell adhesion matrix (data not shown).

## Discussion

One of the most important issues limiting the use of CTCs as an alternative to invasive biopsies has been their relatively low yield. Using the CellSearch System (Veridex), the most widely available technology, the median yield is reported to be in the range of 1 cell per ml of blood screened (Cristofanilli *et al*, 2004). Our results with patients with breast and lung cancer using the CellSearch CEK platform are consistent with that finding. However, two seemingly paradoxical observations prompted us to explore this technology further. The first is that several studies using the CellSearch platform have demonstrated that the instrument was highly efficient at capturing immortalised breast cancer cell lines spiked into the human blood, with yields of ⩾85% of the input cells (Allard *et al*, 2004). We have replicated those studies with similar results; a capture rate of 92% (data not shown). The second observation was the finding that the new CTC–chip microfluidic technology reported mean CTC counts from patients with breast cancer of 79 cells per ml of blood (Nagrath *et al*, 2007). If the CellSearch technology is already highly efficient at capturing CTCs in model systems using cancer cell lines, how can another technology report 10–100-fold higher yields of CTCs from patient samples? We postulated that human CTCs, particularly those that have been subject to anti-cancer treatment, may be significantly more ‘fragile’ than the cancer cell lines used in the initial evaluation of the CellSearch platform. Owing to this fragility, the multiple processing steps involved in the CEK system, including additional washes and labelling steps, could cause degradation and loss of captured CTCs.

We hypothesised that the CPK system, which involves significantly fewer processing steps, may provide improved yields of CTCs from patient samples. Indeed, we observed that the CPK consistently provided a significantly greater yield of CTCs than that obtained with the CEK platform. The median difference was 29-fold in breast cancer samples and 36-fold in lung cancer samples (Figure 1). In addition, virtually all of the breast and lung patients had detectable CTCs using the CPK method, whereas our results with the CEK system demonstrate only 72% of patients had detectable CTCs, consistent with the published results (Hayes *et al*, 2006). The improvement in yield observed with the CPK was not simply due to the differences between the CEK's semi-automated counting system and the CPK's manual counts (Supplementary Figures S1E and F). When the CTCs from both methods were counted manually, the differences persisted. Instead, our observation that when cell lines were pretreated with apoptosis-inducing agents (Figure 4), the yield of CTCs dropped dramatically, suggests that CTC fragility, coupled with the more intensive processing inherent in the CEK method, likely accounts for the observed differences between the CEK and CPK methods.

Importantly, we also found that the purity of the CTCs obtained from the CPK method was quite high, typically in the 60–70% range. This was somewhat unexpected as discussions of the CellSearch system in the literature cite purities in the 0.01–0.1% range, due to contaminating leukocytes. In our studies with the CPK system, the number of contaminating leukocytes, using either patient samples or blood from normal volunteers, and assessed using multiple independent methods, (IF, fluorescence-activated cell sorting), ranged from 20 to 476, and was always less than 25% of total cells. The one study we identified describing the number of contaminating leukocytes observed with the CPK method reported a range of 60–929 CD45-positive cells per sample (Sieuwerts *et al*, 2009). This study did not report the percentage of CTCs *vs* leukocytes in their paper, so their results cannot be compared with our data in that regard.

As has previously been demonstrated with the CEK system, we observed that the CPK method has high intrapatient reproducibility and that blood samples remain stable at room temperature for up to 72 h before sample processing (Allard *et al*, 2004). As many clinical trials involve multiple centres, this sample stability provides the opportunity to collect specimens from multiple sites and analyse them in one central location. This is an advantage over the recently developed microfluidic-based CTC-chip method. That technology has a very low throughput and a requirement for samples to be run within 2 h of being drawn (Nagrath *et al*, 2007). These limitations make it currently incompatible for use in multicentre clinical trials.

The CPK method clearly does not replace the CEK technique's well-validated usefulness as a prognostic tool for clinical samples. Instead, we feel that the CPK technique's relatively high yield, purity, and sample stability make it well suited for use in obtaining CTCs for molecular characterisation, facilitating clinical phenotyping and investigational studies. As an example of the former, we have used the technique to assess the *HER2* gene amplification status of CTCs by FISH and compared it with both the patient's primary breast cancer and tissue from matched distant metastatic sites. In patients with clinically HER2-positive primary and metastatic cancers, virtually all (98%) also had HER2-amplified CTCs. In contrast, in patients with clinically HER2-negative primary cancers, we observed a significant number (33%) of discordant cases in which a patient's CTCs had clear amplification of the *HER2* locus, despite an absence of *HER2* amplification in the primary cancer. This result is consistent with the data from several other small studies demonstrating discordant HER2 expression in CTCs and together provide the rationale for a clinical trial of a HER2-directed therapy in such patients to definitively test the clinical relevance of this observation (Meng *et al*, 2004; Wulfing *et al*, 2006; Pestrin *et al*, 2009). To the best of our knowledge, our study is the first to also examine the relationship between HER2 amplification in CTCs and that of metastatic biopsy tissue. In six out of seven patients with HER2-negative primary cancers and HER2-amplified CTCs, the metastatic biopsy tissue was HER2 negative, matching the primary cancer rather than the CTCs. This observation suggests that either CTCs represent a separate population of cells distinct from that which makes up the bulk of the metastatic disease, or that the cancer developed HER2 amplification in the interval between the patients’ metastatic biopsy and the CTC collection. If the latter explanation were correct, one might expect to see a correlation between the time interval from biopsy to CTC collection and the likelihood that the CTC would gain HER2 amplification. Although we did not observe such a trend (data not shown), our sample size is too small to make any conclusions about the mechanism of this discordance.

In summary, we have demonstrated that using the CellSearch CPK assay, an instrument that is currently available in hundreds of clinical and research laboratories, CTCs can be isolated in sufficient numbers and of sufficient purity to allow for their molecular characterisation. This technology potentially has a large number of applications in investigating the biology of metastatic cancer and in drug development in which it can be used to identify predictive biomarkers, mechanisms of resistance, and facilitate pharmacodynamic studies.

## Figures and Tables

**Table 1:** Relationship between HER2 amplification of primary cancer and that of CTC

**Primary tumor**	**Total No. of patients**	**Patients with HER2−CTCs**	**Patients with HER2+CTCs^a^**	**Discordance**
HER2+	45	1	44	2%
HER2−	30	20	10	33%

Abbreviations: CTC=circulating cancer cells; HER2=human epidermal growth factor receptor 2.

a HER2 positive defined as ratio of HER2/CEP17 ⩾2.0 by FISH.
