# Supplementary material for: Improving the yield of circulating tumour cells facilitates molecular characterisation and recognition of discordant HER2 amplification in breast cancer
Source: Br J Cancer. 2010 May 11;102(10):1495–502. doi: 10.1038/sj.bjc.6605676 (PMC2869174; doi:10.1038/sj.bjc.6605676)
Supplement: Supplementary data [file 6605676x1.ppt]

## Slide 1
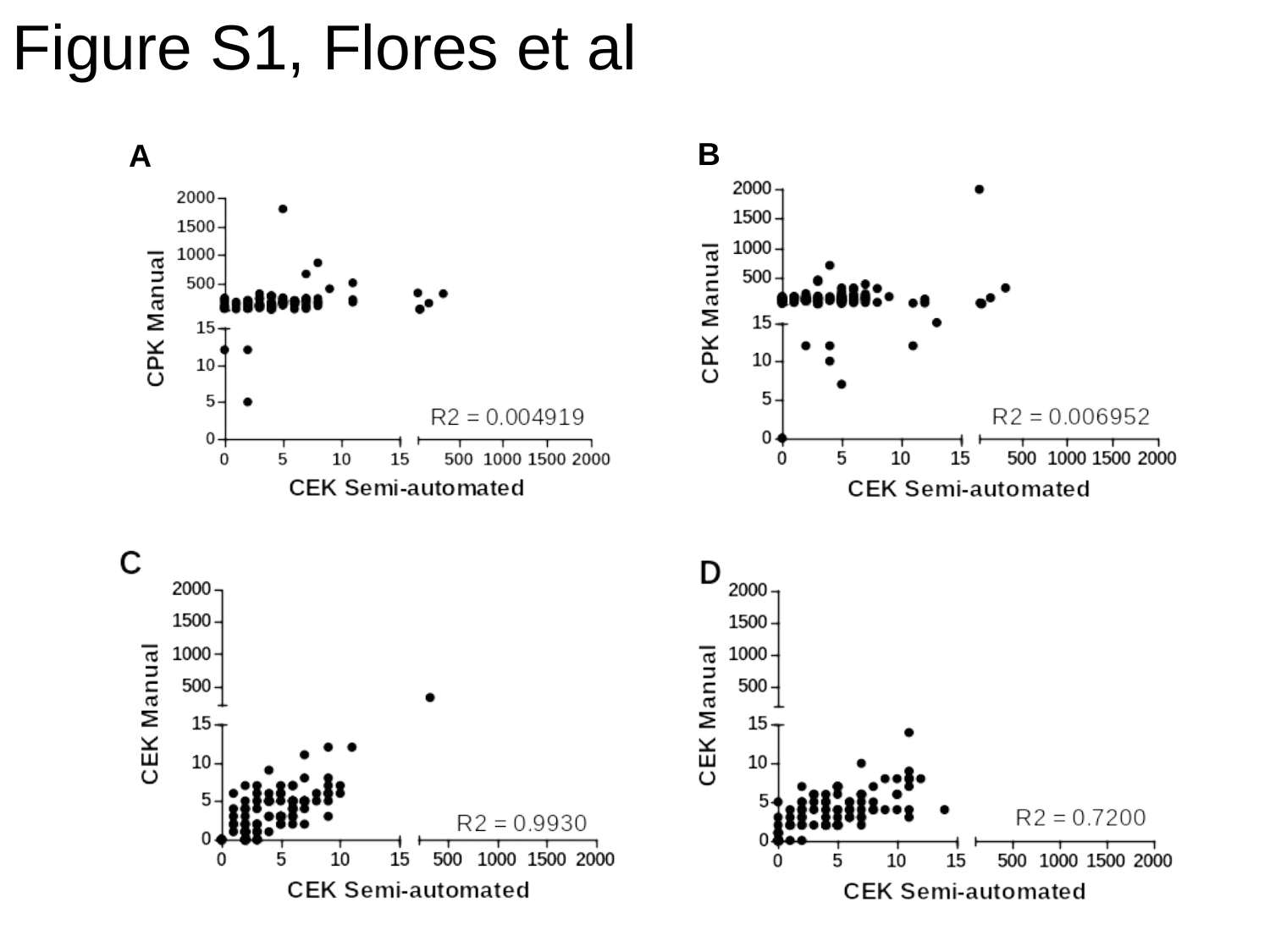

Figure S1, Flores et al
B
A

## Slide 2
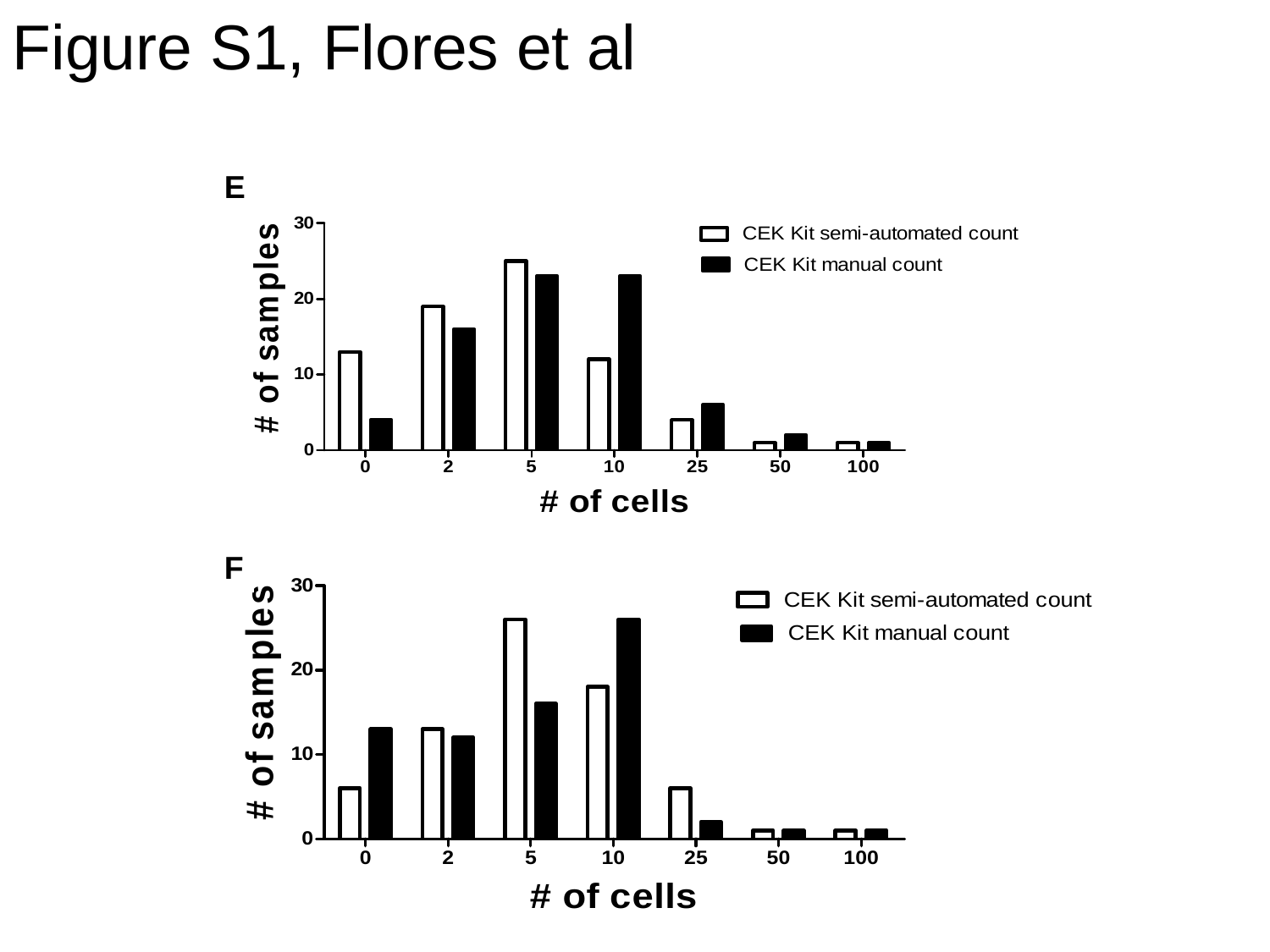

# Figure S1, Flores et al
E
F

## Slide 3
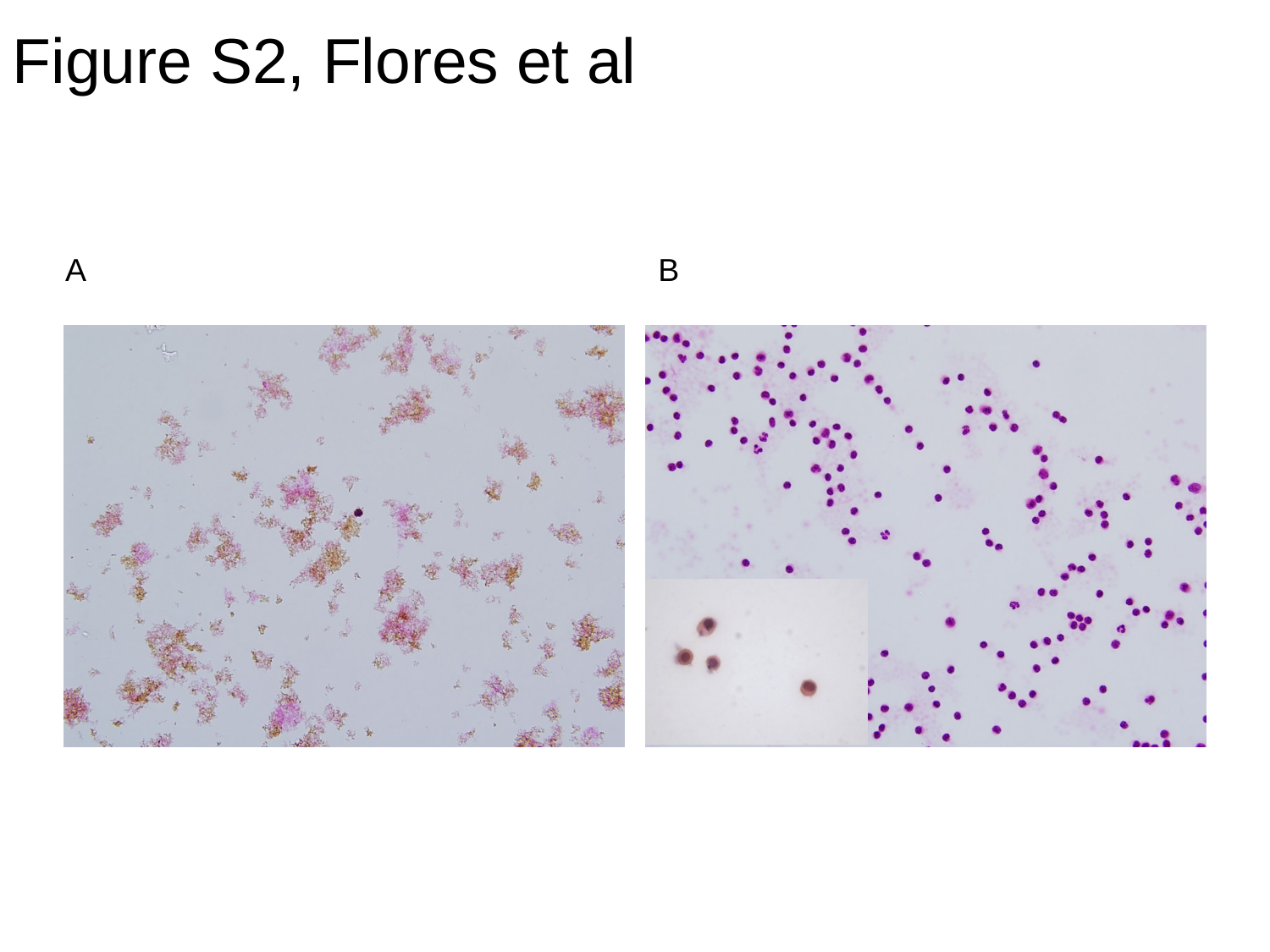

# Figure S2, Flores et al
A
B

## Slide 4
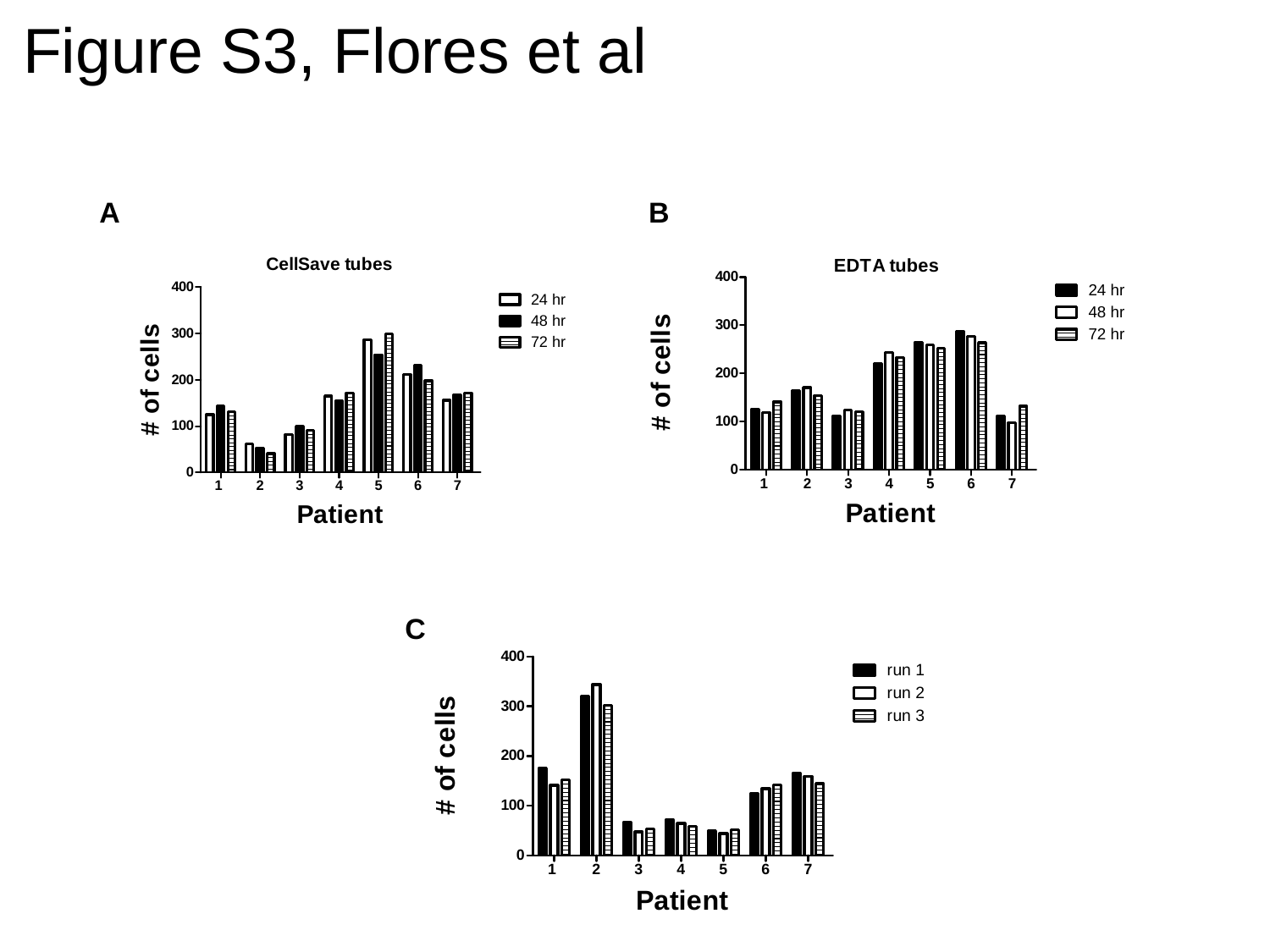

# Figure S3, Flores et al

## Slide 5
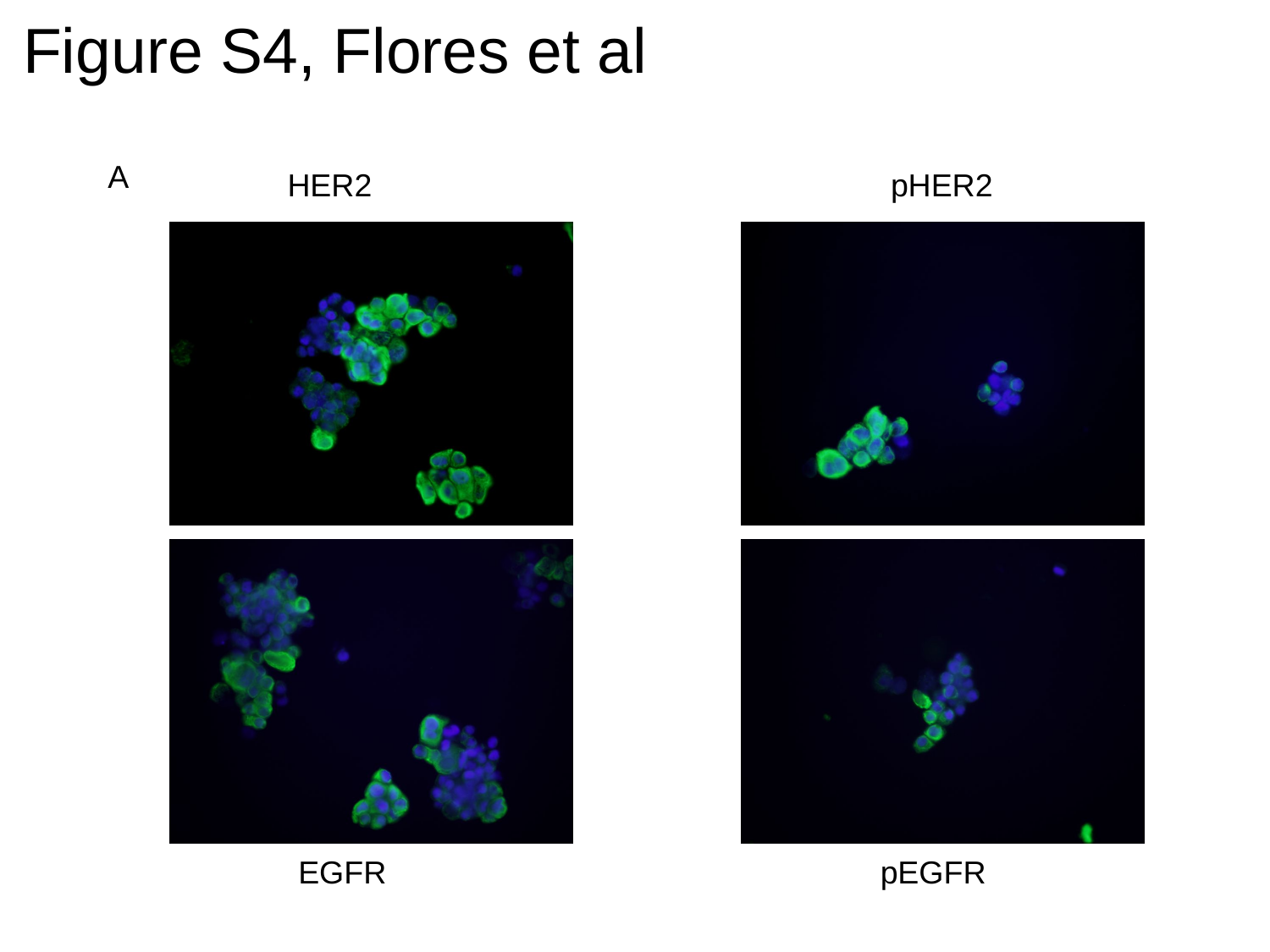

# Figure S4, Flores et al
A
HER2
pHER2
EGFR
pEGFR

## Slide 6
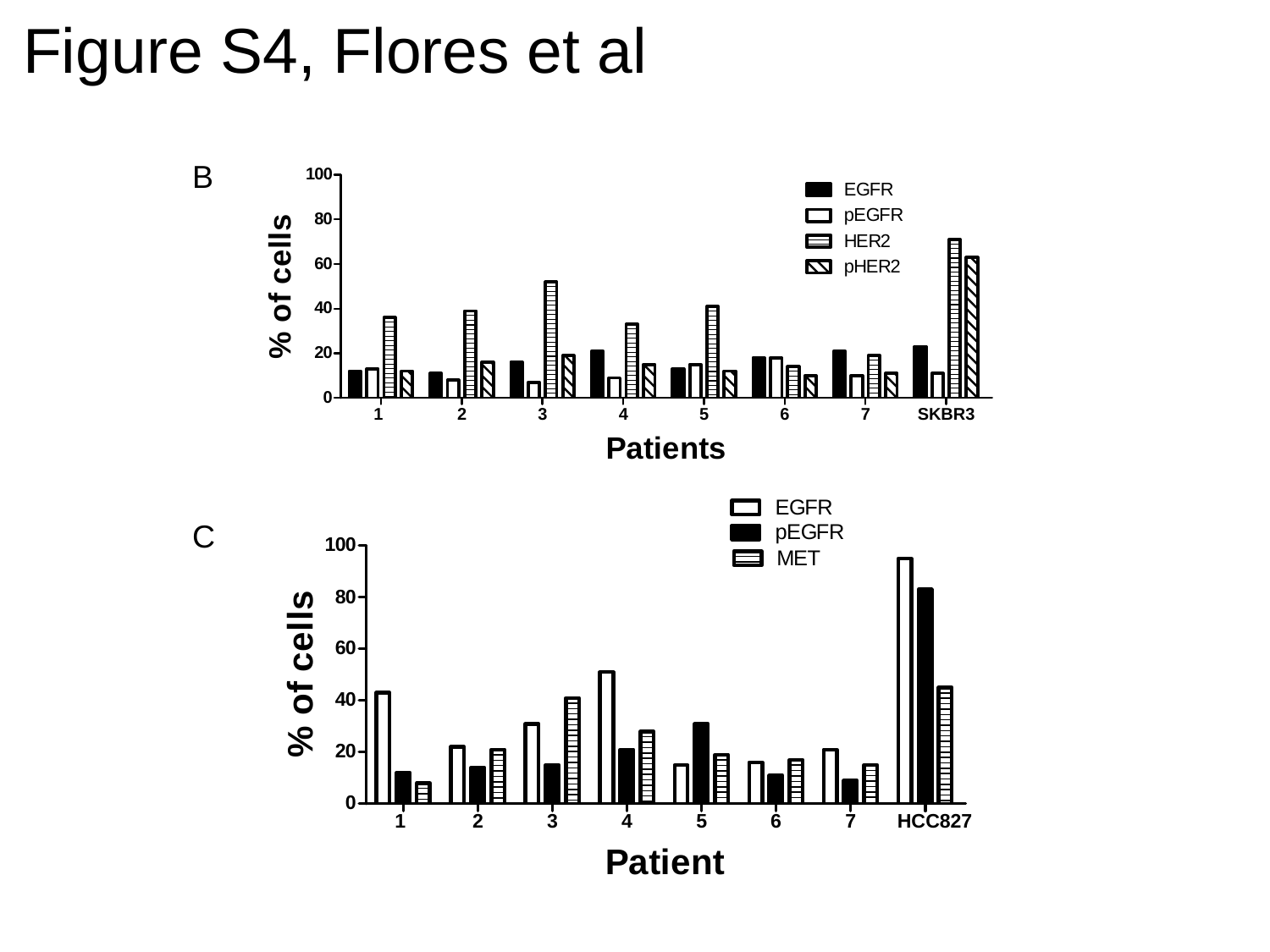

# Figure S4, Flores et al
B
C

## Slide 7
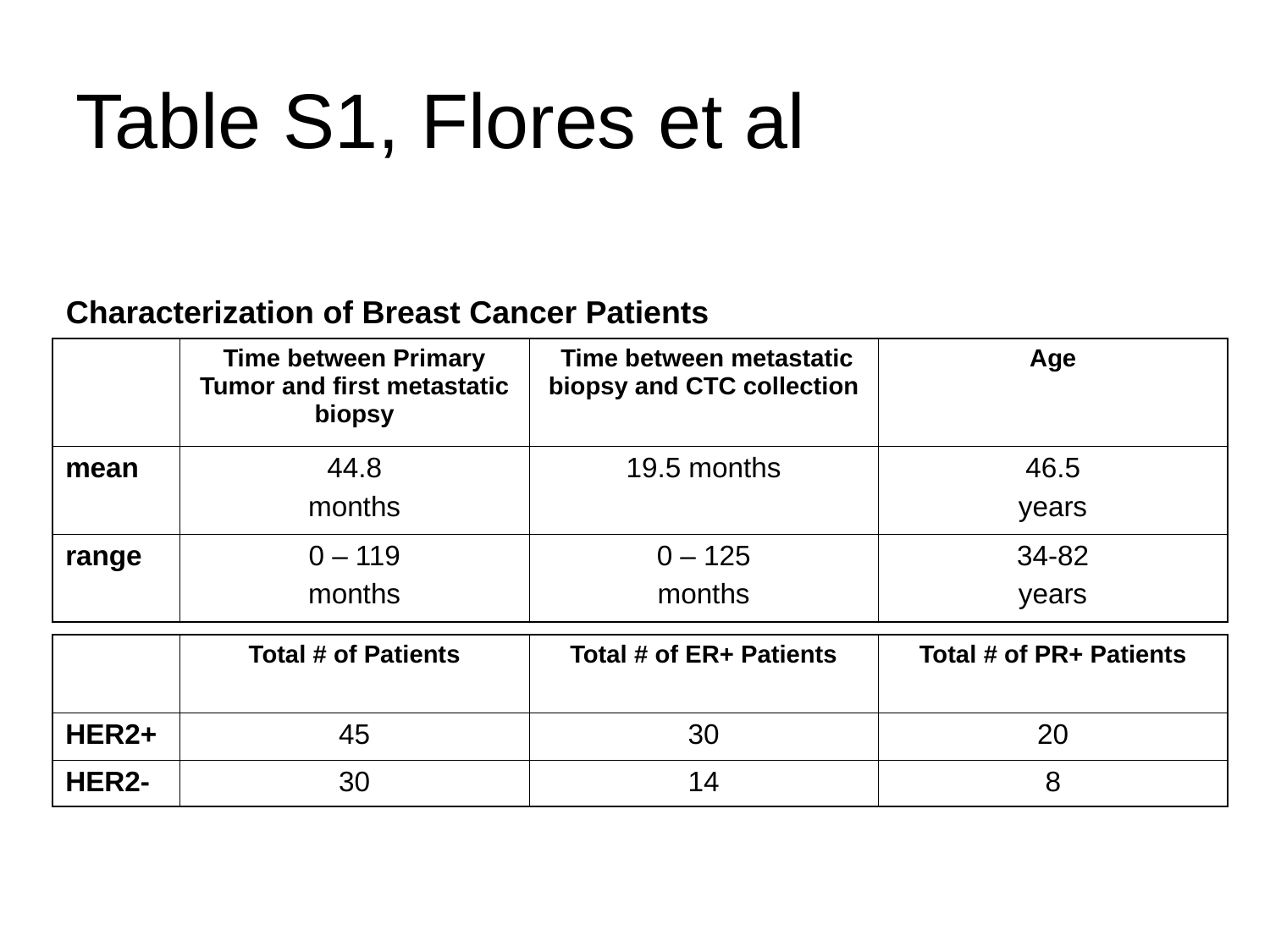

# Table S1, Flores et al
Characterization of Breast Cancer Patients
| | Time between Primary Tumor and first metastatic biopsy | Time between metastatic biopsy and CTC collection | Age |
| --- | --- | --- | --- |
| mean | 44.8 months | 19.5 months | 46.5 years |
| range | 0 – 119 months | 0 – 125 months | 34-82 years |
| | Total # of Patients | Total # of ER+ Patients | Total # of PR+ Patients |
| --- | --- | --- | --- |
| HER2+ | 45 | 30 | 20 |
| HER2- | 30 | 14 | 8 |

## Slide 8
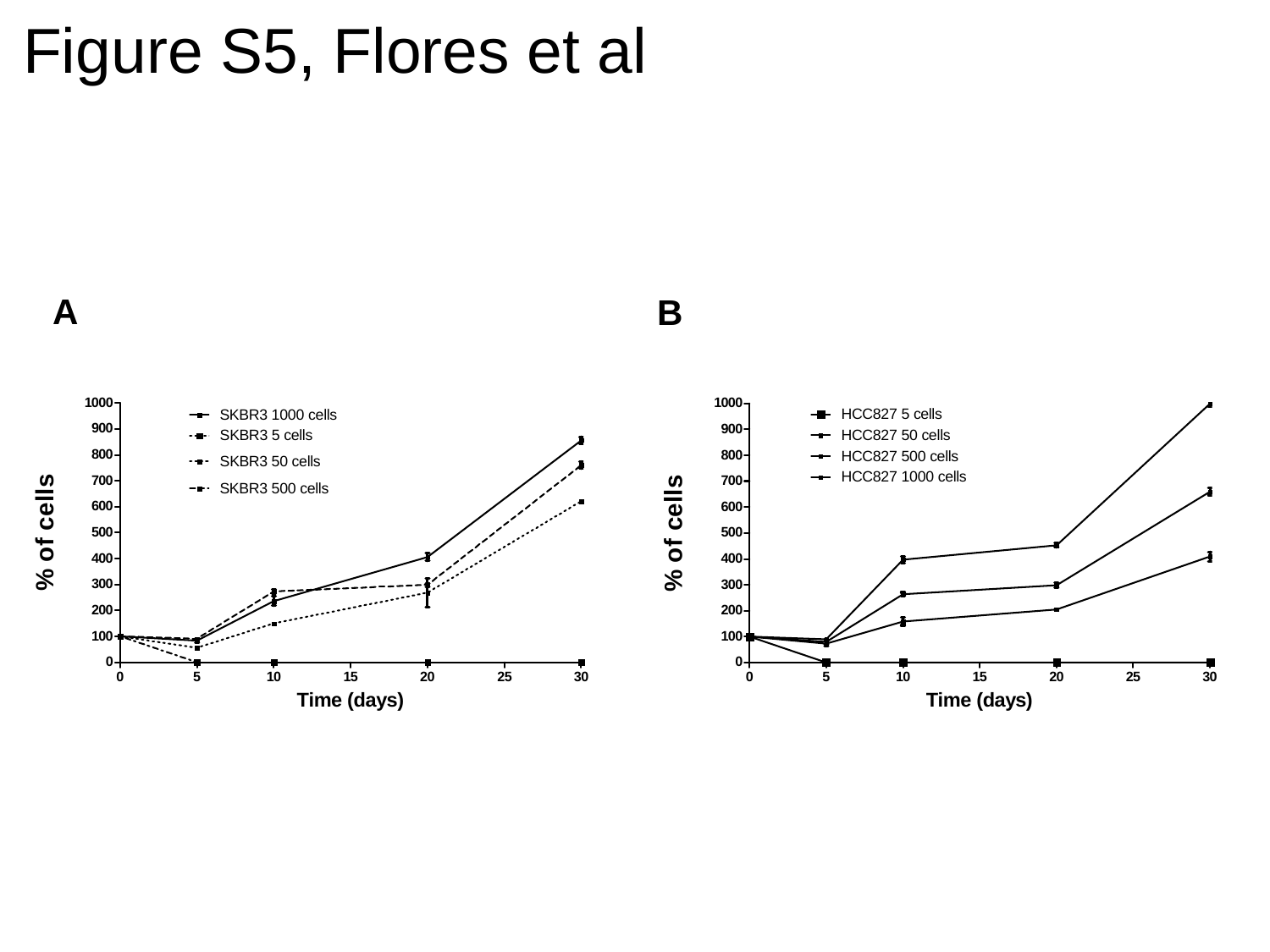

# Figure S5, Flores et al
