# Supplementary material for: Improving the yield of circulating tumour cells facilitates molecular characterisation and recognition of discordant HER2 amplification in breast cancer
Source: Br J Cancer. 2010 May 11;102(10):1495–502. doi: 10.1038/sj.bjc.6605676 (PMC2869174; doi:10.1038/sj.bjc.6605676)
Supplement: Supplementary Methods [file 6605676x2.doc]

**Supplemental Methods**

**Propagation of cell lines processed by CEK method**

For in vitro recovery experiments SKBR3 cells were grown in DMEM with 10%FBS, 10µg/ml EGF, and 1.5mM L-glutamine. HCC287 cells were grown in ACL4 (Invitrogen) with 10% FBS. For further details see Supplemental data. 48-well plates were coated with 100ul of laminin 5 solution (5–7 µg/mL) overnight at 4°C. Cells were grown at 37^o^C in a humidified 10% CO_2_ atmosphere. Cells were either grown as a monolayer, or as a cell suspension in 6-well low adhesive plates (CellGrow). Cells that were grown in cell suspension prior to the isolation process grew better when plated into low adhesion plates for 24 hours after isolation and then plated into laminin 5 or Matrigel coated plates. Cells that were grown as monolayer, prior to the isolation process grew better when they were plated into coated plates immediately after isolation. We observed that it was important for the viability of the adherent cultures to remove dead cells and free ferroparticles and replace the media every 24 hours during the first 7-10 days. Because many cells need more than 24 hours to attach, the removed media should be transferred to freshly coated wells as long as necessary to maximize yields. Suspension cultures should be diluted in a large amount of fresh medium to minimize the toxic effect of dying cells and ferroparticles. Once cells start to proliferate a magnet can be use to remove remaining ferroparticles.

**Analysis of CTC by FISH**

Slides were maintained in a horizontal position throughout the entire hybridization and washing procedure. Briefly, cells were fixed with methanol: acetic acid (3:1), dehydrated in an alcohol series (50%, 75%, 90%, 100%) and air dried. Five microliters of the probe mixture was applied, slides were covered with cover glass, and sealed with rubber cement. Pre-hybridization was done with hybridization buffer without dextran sulfate for 1 hour at 37C. Denaturation was performed at 94^o^C for 5 minutes. Hybridization was done overnight at 37^o^C. Following hybridization, slides were washed in 1x SSC 3 times for 10 minutes each at 37^o^C (maintaining the horizontal position), briefly air dried, and counterstained with Vectashield/DAPI (Vector). Manual quantification of tumor cells was performed by determining the total number of whole nuclei (DAPI-positive signals).

*Immunofluorescence/immunohistochemistry staining*

Cytospin slides were fixed in 4^o^C methanol at for 5 minutes and air dried. Slides were rehydrated in PBS-Tween in a flat position for 5 minutes and then incubated in normal serum block (Vector Laboratories), followed by avidin-biotin block (Vector Laboratories). Slides were incubated with primary antibodies at appropriate dilution for 1 hour at room temperature or overnight at 4 °C, rinsed in PBS-Tween 20 two times for 5 minutes in a horizontal position. Slides were then incubated with biotinylated secondary antibody at appropriate dilution in PBS for 30 minutes at room temperature. Detection was performed with FITC in PBS for 30 minutes at room temperature and counterstained with DAPI with anti-fade mounting medium (Vector) for Phospho- HER2, Phospho-MET, and Phospho-EGFR (Cell Signaling).

Ki-67, TUNEL, and Cyclin D1 immunohistochemistry was carried out similarly to that described above, but with the following modifications. Cytospin slides, after fixation and blocking, were microwaved (in a horizontal position) in citrate buffer for 5 minutes. The slides were allowed to cool for at least twenty minutes, washed three times with TBS-Tween20 and incubated with Ki-67 primary antibody (Dako) for one hour at room temperature. Detection was performed by using biotinylated secondary antibody followed by the ABC peroxidase detection system (Vector) and DAB/methylgreen Counterstain (Vector).

Ficoll-isolated leukocytes were used as a positive control for CD45 staining and a negative control for Cytokeratin staining and the SKBR3 breast cancer cell line were used as a positive control for Cytokeratin staining and negative control for CD45. For these and the other antibodies, appropriate cell line controls were used as well as multiple negative controls (non-specific first antibody, no specific antibody, secondary antibody of the wrong species, and no detection enzyme).
