# Supplementary material for: Improving the yield of circulating tumour cells facilitates molecular characterisation and recognition of discordant HER2 amplification in breast cancer
Source: Br J Cancer. 2010 May 11;102(10):1495–502. doi: 10.1038/sj.bjc.6605676 (PMC2869174; doi:10.1038/sj.bjc.6605676)
Supplement: Supplementary Figure Legends [file 6605676x3.doc]

**Supplemental Figure Legends**

**Figure S1** *CTC counts on a per patient basis*. CTC counts from patients with **A)** breast cancer or **B)** NSCLC using the CEK/semi-automated quantification method vs. CPK/manual quantification method. CTC counts from patients with **C)** breast cancer or **D)** NSCLC using the CEK/semi-automated quantification method vs. CEK /manual quantification method. *Differences in cell yields are not due to the method of enumeration.* Samples from patients with breast cancer (n = 75) (**E)** or NSCLC (n = 71) **(F)** processed by the CEK method were analyzed by semi-automated quantification (open columns) followed by manual quantification (hatched columns).

**Figure S2**. **A)** Representative photo of a blood sample processed with the CPK method from a healthy volunteer **B)** Blood processed with CPK method from a patient with breast cancer. Both samples were stained with hematoxylin and eosin.

**Figure S3.** *The CPK method has high intrapatient reproducibility and samples are stable over 72 hour.*  The plots depict CTC yields from paired samples from 7 patients with NSCLC collected in **A)** CellSave^TM^ or **B)** EDTA tubes and incubated at room temperature for 24-72 hrs prior to processing by the CPK method. **C)** Depicts variability of CTC yields from triplicate samples from 7 patients with NSCLC collected at the same time point and processed in parallel by the CPK method**.**

**Figure S4.** *CTCs are suitable for molecular characterization* **A)** Representative immunofluorescence images of CPK-processed CTCs from patients with HER2+ breast cancer (top panels) and NSCLC (lower panels) labeled with DAPI and individually for anti- HER2, pHER2, EGFR, and pEGFR. **B)** Percentage of CTCs from patients with HER2+ breast cancer and the cell line SKBR3, or **C)** with NSCLC and the cell line HCC827 that stain with the indicated antibodies.

**Figure S5.** *CTCs captured by the CPK method are viable for in vitro propagation*. The indicated number of the **A)** Breast cell line SKBR3 or **B)** NSCLC line HCC827 were spiked into normal blood without fixative, captured with the CPK method, then grown in culture on laminin coated plates. The plots depict the number of viable cells at the given time after plating as a percentage of the initial number of cells spiked into blood (±1 SD).
